# Treadmill Exercise Training Improves Vascular Endothelial Growth Factor Expression in the Cardiac Muscle of Type I Diabetic Rats

**DOI:** 10.14740/cr314w

**Published:** 2014-02-27

**Authors:** Nour S. Erekat, Muhammed D. Al-Jarrah, Ahed J. Al Khatib

**Affiliations:** a Department of Anatomy, Faculty of Medicine, Jordan University of Science and Technology (JUST), Irbid, Jordan; b Department of Rehabilitation Sciences, Faculty of Applied Medical Sciences, JUST, Irbid, Jordan; c Department of Pathology, Faculty of Medicine, JUST, Irbid, Jordan

**Keywords:** Type I diabetes, VEGF, Cardiac muscle

## Abstract

**Background:**

Vascular endothelial growth factor (VEGF) expression is a potent mitogen for endothelial cells that is involved in angiogenesis. Cardiac VEGF is decreased in many pathologic conditions, including diabetes mellitus and aging. Exercise training has improved VEGF expression in the aging heart. Thus, the aim of our study is to illustrate the impact of treadmill exercise training on the cardiac VEGF expression in type I diabetic rats.

**Methods:**

Twenty normal Sprague-Dawley rats and Sprague-Dawley rats with streptozotocin-induced diabetes were divided into the following equal groups: sedentary control (SC), exercised control (EC), sedentary diabetic rats (SD) and exercised diabetic rats (ED). Immunohistochemistry was used to investigate VEGF expression in the cardiac tissue in each of the four different groups.

**Results:**

Cardiac VEGF expression was significantly (P < 0.05) lower in SD compared with that in SC. However, exercise training significantly (P < 0.01) enhanced VEGF expression in the cardiac tissue in ED compared with that in SD.

**Conclusion:**

Our present data suggest that treadmill exercise training improved diabetes-induced downregulation in the cardiac VEGF expression.

## Introduction

Vascular endothelial growth factor (VEGF) is a potent mitogen for endothelial cells that is involved in blood vessel formation, a process called angiogenesis [1-4]. VEGF is also involved in vasodilation and antiapoptosis [5-10]. Alterations in VEGF expression have been demonstrated in many pathologic conditions [11, 12]. For instance, although VEGF upregulation has been shown in Parkinson disease substantia nigra and in the diabetic retinal and renal tissues [13-22], its downregulation has been demonstrated in diabetic skeletal muscle [23-25].

VEGF has been implicated in the pathogenesis of many heart diseases, such as coronary artery disease, ischemic heart disease and strokes [11, 26-29]. Diabetes mellitus is a major risk factor for cardiovascular disorders, including coronary heart disease, stroke, peripheral arterial disease and cardiomyopathy. Such cardiovascular complications significantly increase the risk for morbidity and mortality in diabetic patients [30-35]. Decreased VEGF level has been reported in diabetic cardiac tissue and it has been suggested to cause the impaired collateral formation, which is probably accounting for the increased risk for morbidity and mortality in patients with diabetes mellitus [36-39]. Furthermore, normalization of the downregulated myocardial VEGF level is suggested to improve cardiac dysfunction in diabetes [40].

Exercise training has been shown to exert beneficial effect on VEGF expression in pathologic conditions [23, 41, 42]. For example, downregulated VEGF in the skeletal muscle of diabetic patients was enhanced by exercise training [23]. Similarly, exercise training could improve the decreased level of VEGF in the aging heart [41]. Moreover, treadmill exercise has increased the expression of VEGF in the brain of chronic Parkinsonian mice [42].

To our knowledge, the impact of treadmill exercise training on VEGF expression has never been investigated in diabetic cardiac tissue. Therefore, using immunohistochemistry and light microscopy, the purpose of this study was to examine the effect of treadmill exercise training on VEGF expression in the heart from rats with streptozotocin-induced diabetes mellitus.

## Materials and Methods

### Animals

Forty Sprague-Dawley rats were used in this study and randomly divided into four equal groups. Sedentary control (SC, n = 10), exercised control (EC, n = 10), sedentary diabetic (SD, n = 10) and exercised diabetic (ED, n = 10). Animals were housed in individual cages at 22 ± 1 °C in a controlled room with a 12:12 light:dark cycle. The animals were allowed free access to standard chow and water. Animal care and experiments were performed in accordance with the research committee guidelines for animal experimentation at Jordan University of Science and Technology. Alloxan (120 mg/kg) was intraperitoneally injected in the rats in the two diabetic groups. Simultaneously, intraperitoneal saline (120 mg/kg) injections were given to the rats in the two control groups. Three days later, successful induction of diabetes was confirmed by detecting hyperglycemia in the rats, which had fasting blood glucose above 250 mg/dL.

### Exercise protocol

The rats were exercised according to the exercise training protocol previously described and suggested to provide adequate systemic and cellular adaptations with this level of aerobic exercise [43]. Briefly, using a custom tredmill with 8 separate lanes, rats in the two exercised, both control and diabetic, groups were running at a speed of 18 m/min, 40 min/day for 5 days/week. Although sedentary rats did not exercise, they were transported daily to the training room, in order to expose them to the same environment as the exercised groups of animals.

### Immunohistochemistry of VEGF in the heart

Tissues were collected from the left ventricle of the heart and fixed in 4% paraformaldehyde. Then, 5 µm thick paraffin-embedded sections were prepared. After that, the sections were processed via immunohistochemistry according to the protocol described previously [42]. Briefly, the sections were deparaffinized in xylene for 2 minutes twice, and subsequently rehydrated through consecutively descending dilutions of alcohol (100%, 90%, 80% and 70%) ending in water (2 minutes each step). After that, the sections were treated in the reveal solution (RV1000M, Biocare Medical, Concord, CA) in the Decloaking chamber (Biocare Medical) for 2 minutes. After cooling sections down to room temperature, endogenous peroxidase activity was blocked by incubating the sections with 3% hydrogen peroxide in methanol for 5 minutes. After washing the sections in phosphate buffered saline (PBS), they were incubated with anti-VEGF antibody (Santa Cruz Biotechnology, Santa Cruz, CA), diluted according to vendor instructions, for 1 hour at room temperature. Subsequently, sections were washed in PBS and incubated with biotinylated secondary antibody (LSAB kit, Dako Carpinteria, CA) for 15 minutes at room temperature, then washed with PBS. Sections were incubated with streptavidin horse radish peroxidase (LSAB kit, Dako) for 15 minutes at room temperature and washed with PBS. Finally, 3, 3’-Diaminobenzidine (DAB) substrate was applied for 2 minutes or longer, until the desired color intensity was developed, and then the slides were washed with tap water to stop the reaction. Negative control slides were processed without the primary antibody. All sections were counterstained with hematoxylin and viewed under the light microscopy. Ten slides from each animal group were evaluated for VEGF expression in the left ventricle.

### Data collection and analysis

The sections were photographed with digital camera. Ten slides from each animal of all 10 animals in each of the 4 groups were analyzed by counting the total pixels area occupied by positive staining, using Adobe Photoshop software, as described previously [42, 44]. VEGF expression was analyzed, in the cardiac tissue from the different groups, and statistically compared among the 4 different groups using one way ANOVA followed by independent sample t-test. Differences in VEGF expression were considered statistically significant at P value < 0.05.

## Results

**Figure 1:**
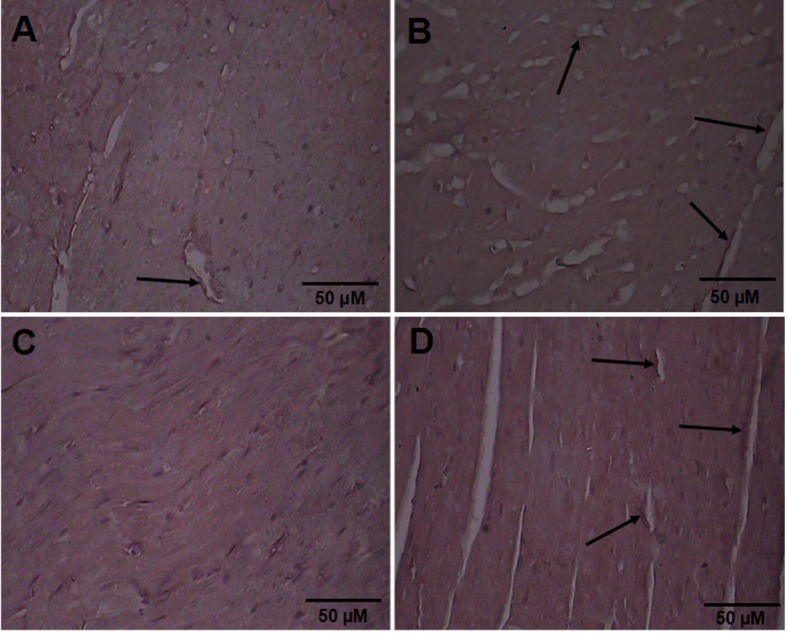
Immunohistochemical staining of VEGF cardiac tissue 5 µm thick paraffin-embedded sections. VEGF immunoreactivity (at the tips of the arrows) is stronger following exercise training in the diabetic rats. A) From SC. B) From EC. C) From SPD. D) From EPD. SC: Sedentary Control. EC: Exercised Control. SD: Sedentary diabetic. ED: Exercised diabetic.

Immunohistochemical staining revealed that there was evidence of VEGF in control hearts (Fig. 1A). Moreover, VEGF immunoreactivity was found in the heart from exercised controls (Fig. 1B). On the other hand, immunohistochemical staining barely showed VEGF expression in diabetic heart (Fig. 1C). In contrast, VEGF immunoreactivity was observed in the hearts from exercised diabetic rats (Fig. 1D).

**Figure 2:**
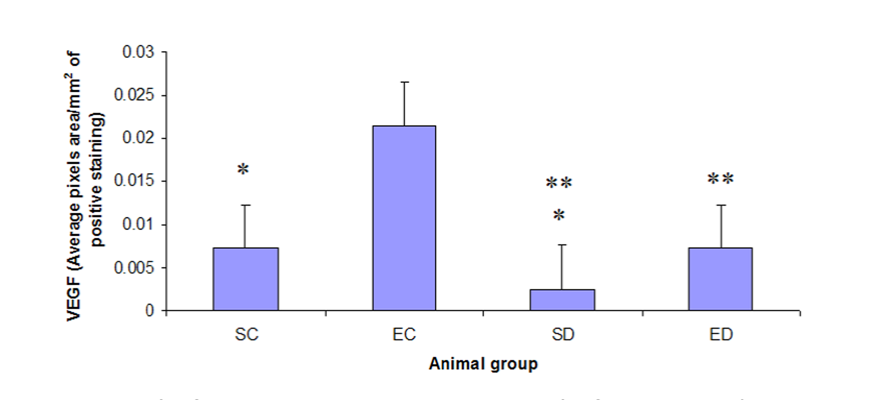
Expression of VEGF in the cardiac muscle. The expression level of VEGF decreased significantly in the diabetic sedentary group compared to sedentary control groups (P < 0.05*). Exercise training significantly increased the expression level of VEGF in diabetic rats (P < 0.01**). SC: Sedentary Control. EC: Exercised Control. SD: Sedentary Diabetic. ED: Exercised Diabetic.

VEGF expression in the diabetic heart is statistically significantly lower (P < 0.05) than in that in the control heart (Fig. 2). However, treadmill exercise training has statistically insignificantly increased (P > 0.05) cardiac VEGF expression in the control group (Fig. 2). On the other hand, cardiac VEGF expression is statistically significantly increased (P < 0.01) in the exercised diabetic group when compared with that in the sedentary diabetic group (Fig. 2).

## Discussion

This is the first study to report the impact of treadmill exercise training on VEGF expression in the heart of rats with streptozotocin-induced diabetes mellitus. Our analysis reveals that treadmill exercise training upregulated VEGF expression in the cardiac muscle of diabetic rats.

We could detect VEGF in the heart of control rats (Fig. 2). VEGF has been shown to be expressed in the normal heart [45]. VEGF mediates angiogenesis in both physiologic and pathologic conditions [1, 45-47]. Besides, VEGF has many endothelial cell actions relating to permeability, vasodilation and antiapoptosis [5-10, 48-53]. Our results reveal a statistically insignificant increase in the VEGF expression in the cardiac muscle of the controls following treadmill exercise training (Fig. 2). Thus, our study confirms the previous report demonstrating insignificant change in VEGF mRNA and protein in the heart from normal rats in response to exercise [54, 55]. On the other hand, VEGF protein expression was shown to significantly increase 1 day after exercise training in intact mice and gradually return to baseline after 4 days [56].

The present study suggests that the induction of diabetes mellitus by streptozotocin has decreased the expression of VEGF in the heart (Fig. 2). Our results are consistent with the previous reports, which have shown VEGF downregulation in the heart in diabetic and insulin-resistant states and assumed the loss of insulin-induced VEGF expression as the underlying mechanism [36, 57, 58]. In addition to that, those previous reports have suggested decreased level of cardiac VEGF and the consequent inadequate collateral formation as a potential molecular explanation for the increased risk of cardiovascular morbidity and mortality in patients with insulin resistance and diabetes [36, 40, 59].

Exercise has been suggested to have a beneficial role on the risk of coronary heart diseases in diabetic patients [60]. To examine the mechanism by which exercise ameliorates cardiovascular outcome in diabetics, we investigated the effect of treadmill exercise training on the cardiac VEGF expression in rats with streptozotocin-induced diabetes mellitus. Downregulation of VEGF expression has been demonstrated in the aging heart [41]. However, VEGF expression has been improved in the aging heart following endurance exercise training [41]. Similarly, our results (Fig. 2) reveal elevated cardiac VEGF levels in rats with streptozotocin-induced diabetes mellitus following treadmill exercise training. The elevated level of cardiac VEGF, shown by our study (Fig. 2), may explain the increased collateral development and improved endothelial vasodilation in the diabetic heart following exercise training demonstrated in previous studies [61-64].

In conclusion, this is the first study to report the impact of treadmill exercise training on VEGF expression in heart from rats with streptozotocin-induced diabetes mellitus. In summary, treadmill exercise training improves streptozotocin-induced diabetes mellitus-induced downregulation of VEGF expression in the heart.
